# Organic vs. Conventional Milk: Uncovering the Link to Antibiotic Resistance in *Bacillus cereus* sensu lato

**DOI:** 10.3390/ijms252413528

**Published:** 2024-12-17

**Authors:** Marek Bartoszewicz, Urszula Czyżewska, Monika Zambrzycka, Izabela Święcicka

**Affiliations:** 1 Department of Microbiology, Faculty of Biology, University of Białystok, 1J Konstanty Ciołkowski Street, 15-245 Białystok, Poland; urszula.czyzewska@uwb.edu.pl (U.C.); m.zambrzycka@uwb.edu.pl (M.Z.); izabelas@uwb.edu.pl (I.Ś.); 2 Laboratory of Applied Microbiology, Faculty of Biology, University of Białystok, 1J Konstanty Ciołkowski Street, 15-245 Białystok, Poland

**Keywords:** *Bacillus cereus* sensu lato, antibiotic resistance, enterotoxicity, organic milk, conventional milk, food safety, milk microbiology

## Abstract

*Bacillus cereus* sensu lato (*B. cereus s.l.*) comprises mesophilic and psychrotolerant bacteria commonly found in natural environments as well as in organic and conventional milk. Due to their potential toxigenicity and antibiotic resistance, these bacteria pose a significant threat to consumer health. Organic milk production, which prohibits the use of antibiotics and artificial additives, may influence the composition of microbiota between milk types. This study aimed to compare the antibiotic resistance profiles and enterotoxic potential of *B. cereus s.l.* isolates from organic and conventional milk. The results indicate that, although conventional milk contains on average 3 times fewer *B. cereus s.l.* isolates, it has 10–15% more resistant isolates to selected beta-lactams, macrolides, and aminoglycosides. Regarding drug resistance, 21% of *B. cereus s.l.* isolates were multidrug-resistant, and as many as 42% were non-susceptible to two classes of antibiotics. Even among the sensitive isolates, bacteria from conventional milk exhibited on average 2.05 times higher MICs (minimal inhibitory concentrations) for beta-lactams, 1.49 times higher for erythromycin, 1.38 times higher for vancomycin, and 1.38 times higher for azithromycin. Antibiotic resistance was mostly associated with the origin of the isolates. These findings underscore the need for ongoing monitoring of antibiotic resistance and enterotoxicity among opportunistic *B. cereus s.l.* strains, which may pose challenges for public health and veterinary medicine. The results highlight that selective pressure associated with antibiotic use can drive resistance development in bacteria that are not the primary targets of antimicrobial therapy.

## 1. Introduction

*Bacillus cereus* sensu lato (further *B. cereus s.l.*) represents a complex group of spore-forming, facultative anaerobic, Gram-positive bacilli with biofilm formation ability. These bacteria are capable of breaking down a wide range of substrates, often even at relatively low temperatures, making them a key area of interest in food microbiology [1]. Notable representatives of this group include *B. cereus* sensu stricto (further *B. cereus*), *Bacillus thuringiensis*, *Bacillus mycoides*, *Bacillus weihenstephanensis*, *Bacillus wiedmannii*, and *Bacillus anthracis*. However, the taxonomy of this group remains contentious, particularly regarding the classification of individual members within *B. cereus s.l.* as distinct species. For example, *B. thuringiensis* is almost indistinguishable ecologically and biochemically from *B. cereus*, aside from its ability to synthesize parasporal crystal proteins active against insect pests [2]. Recent data suggest that *B. weihenstephanensis* should be considered a variant within *B. mycoides* [3,4,5]. Consequently, in the present work, we will analyze *B. thuringiensis* and *B. cereus* together. *B. mycoides* are psychrotrophic bacilli that form unusual rhizoidal colonies on agar surfaces, while *B. weihenstephanensis* produces regular colonies on solid media. Despite these differences in colony morphology, the two species share similar ecological traits and are nearly identical at the genetic level. Following the recommendation of Liu and colleagues, we will refer to them collectively as *B. mycoides*/*B. weihenstephanensis* [6]. *B. cereus s.l.* is widely distributed in the natural environment, including soil, freshwater, and marine habitats [7], as well as the gastrointestinal tracts of invertebrates and vertebrates [8]. Consequently, it can easily contaminate food products, either through raw materials or via recontamination during food processing [9]. These bacteria can pose significant challenges in food production and storage. Their role in dairy and vegetable production has also been emphasized [7,10].

*B. cereus s.l.* harbors numerous genes associated with potential virulence, such as the *nhe* and *hbl* operons encoding the three-partial non-hemolytic enterotoxin (Nhe) and hemolysin BL (HBL), respectively, and the *cytK* gene facilitating the production of cytotoxin K. Intestine infections involving strains that can biosynthesize these toxins may result in diarrheal syndrome, characterized by diarrhea, weakness, abdominal pain, and nausea, typically manifesting around 24 h after ingestion of contaminated food. Moreover, *B. cereus* is also known for the synthesis of cereulide, facilitated by the *ces* operon. The cereulide intoxication leads to severe vomiting and, in extreme cases, irreversible liver damage, causing even death [4,11].

Antibiotic resistance among bacteria is well-documented, especially concerning significant pathogens such as staphylococci, streptococci, and *Enterobacteriaceae*. However, emerging data indicate that *B. cereus* and related species can also develop antibiotic resistance, posing potential health risks to both humans and animals and serving as a reservoir of resistance genes for other microorganisms [12,13,14]. The findings that isolates from animal-derived foods, such as raw milk, can tolerate higher levels of antibiotics compared to those from natural environments or plant-derived foods are particularly alarming [15]. For *Bacillus* species, penicillin, ampicillin, imipenem, meropenem, vancomycin, amikacin, gentamicin, erythromycin, tetracycline, ciprofloxacin, levofloxacin, clindamycin, chloramphenicol, and rifampicin are most commonly analyzed. On the other hand, resistance in *B. cereus s.l.* is frequently observed against beta-lactams, macrolides, tetracyclines, and aminoglycosides, which are among the most commonly used antibiotics in human and veterinary medicine [16]. The risk associated with the exposure of opportunistic bacteria to frequent subclinical levels of antibiotics used in the veterinary treatment of animals, such as cows, cannot be underestimated and requires further investigation. There is growing concern that under such conditions, continued selective pressure may lead to the development of resistance to other drugs as well.

Organic products, due to the absence of synthetic additives and fertilizers, may differ microbiologically from the respective conventional ones [17]. However, the literature lacks definitive data on the impact of organic farming on the presence of potential pathogens in food products. Organic production imposes restrictions on the use of additives and growth promoters. The use of antibiotics and chemical additives is also prohibited, suggesting that the microflora of organic products may exhibit a lower tolerance to common antibiotics than from conventional food. We hypothesized that organic animal husbandry and the principles of minimizing antibiotic use in organic farming should lead to a lower prevalence of resistant strains. Therefore, we deemed it essential to study *B. cereus s.l.* in milk from conventional and organic farms. This study aims to examine the presence of *B. cereus* and related bacteria in organic food and compare the antibiotic resistance and toxicity profiles of strains found in analogous conventional products.

## 2. Results

### 2.1. Bacillus cereus s.l. Presence in Conventional and Organic Milk

Using membrane ultrafiltration, it was possible to determine the concentration of *B. cereus s.l.* spores in the analyzed samples and to isolate pure bacterial cultures. Based on the number of colonies formed on the surface of nitrocellulose filters, it was established that the mean concentration of bacterial spores in conventional and organic milk differed significantly (Student’s *t*-test, t = −2.59, *p* = 0.032). The values obtained for conventional milk at individual sampling points were as follows: 220 CFU/L (colony forming units/liter), 300 CFU/L, 160 CFU/L, and 280 CFU/L. For organic milk, the corresponding values at the sampling points were: 400 CFU/L, 450 CFU/L, 600 CFU/L, and 1350 CFU/L. Thus, it can be concluded that the mean concentration of spores in conventional milk (from five independent samples from each of four farms) was nearly three times lower (240 CFU/L) than in organic milk (700 CFU/L). Notably, the contamination level in conventional milk was less variable, whereas in organic milk, contamination varied significantly depending on the farm. However, in all cases, organic milk exhibited a higher level of B. cereus *s.l*. contamination compared to its conventional counterpart. Across all samples, *B. cereus*/*B. thuringiensis* were most frequently isolated.

Among the collected bacteria, two isolates did not exhibit hemolysis on blood agar, which is typical for members of this group, except for B. anthracis. However, PCR analyses using primers specific to pXO-1 and pXO2 yielded negative results, and these isolates were also classified as *B. cereus*/*B. thuringiensis*.

Out of the isolated strains, a total of 200 *B. cereus s.l.* isolates, randomly selected from raw milk collected in the Northeastern region of Poland, were analyzed. Among the studied bacteria, there were 120 isolates of *B. cereus*/*B. thuringiensis* (60 from organic milk and 60 from conventional milk) and 80 isolates of *B. mycoides*/*B. weihenstephanensis* (40 from each type of milk). The proportions of the individual taxa reflect their representation among all isolates.

### 2.2. Antibiotic Resistance in B. cereus s.l. from Conventional and Organic Milk

The disk diffusion method was applied to assess the prevalence of *B. cereus s.l.* isolates resistant to commonly used antibiotics, and determining the percentage of strains resistant to selected antibiotics. Cumulative data for all isolates obtained from both types of milk are summarized in Table A1 (Appendix A).

**Figure 1:**
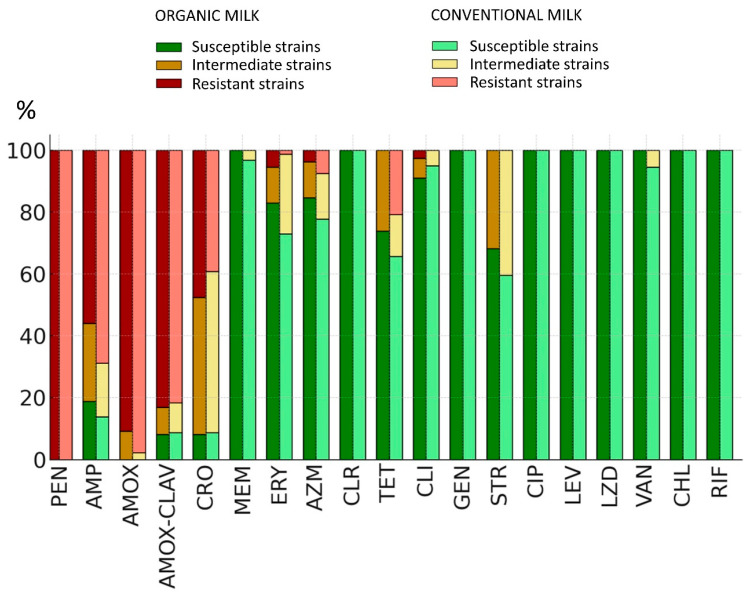
The percentage of antibiotic-resistant, intermediate, and sensitive strains among *B. cereus s.l.* isolated from organic milk (left side of each bar) and conventional milk (right side of each bar). The following abbreviations were used for clarity: PEN, penicillin; AMP, ampicillin; AMOX, amoxicillin; AMOX-CLAV, amoxicillin with clavulanic acid; CRO, ceftriaxone; MEM, meropenem; CHL, chloramphenicol; VAN, vancomycin; ERY, erythromycin; AZM, azithromycin; CLR, clarithromycin; TET, tetracycline; CLI, clindamycin; GEN, gentamicin; STR, streptomycin; CIP, ciprofloxacin; LEV, levofloxacin; LZD, linezolid; RIF, rifampicin.

High resistance levels were observed against certain beta-lactams, such as penicillin and amoxicillin, with over 80% of isolates showing resistance to these agents. In contrast, 100% sensitivity was recorded for antibiotics such as clarithromycin, gentamicin, linezolid, levofloxacin, chloramphenicol, rifampicin, and ciprofloxacin. The highest proportion of intermediate-sensitivity strains was observed for ceftriaxone, streptomycin, ampicillin, tetracycline, and erythromycin. These findings highlight the variability of resistance patterns among *B. cereus s.l.* strains. A comparison of the proportion of resistant, intermediate, and sensitive isolates in organic vs. conventional milk is shown in Figure 1. Interestingly, in nearly all cases, the proportion of sensitive bacteria is higher among isolates from organic milk, except the sensitivity to clindamycin, where the difference is minimal at 4%. Additionally, no significant differences in the frequency of resistant and intermediate strains were observed between *B. cereus*/*B. thuringiensis* and *B. mycoides*/*B. weihenstephanensis*.

### 2.3. Antibiotic Minimum Inhibitory Concentrations (MIC)

**Figure 2:**
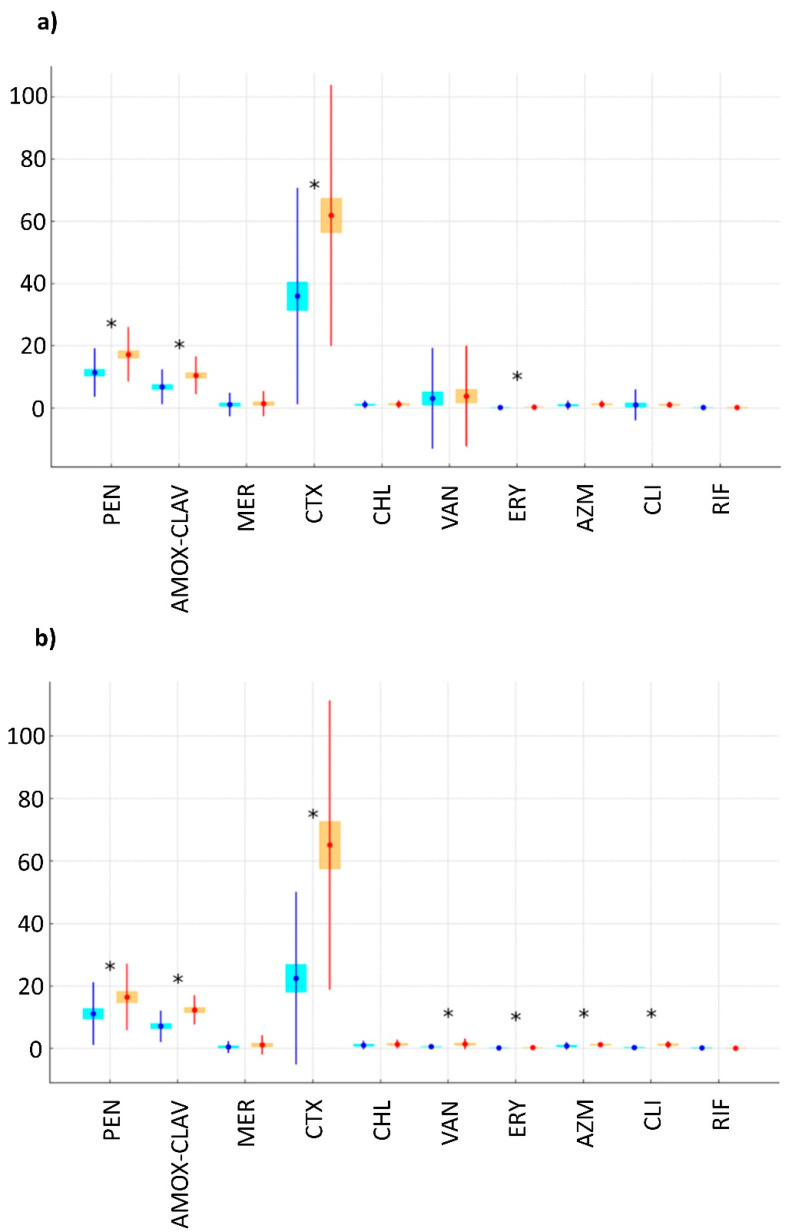
Comparison of mean MIC values of selected antibiotics for *B. cereus*/*B. thuringiensis* (**a**) and *B. mycoides*/*B. weihenstephanensis* (**b**) isolates from organic (blue) and conventional (red) milk. Mean values are represented as points, standard deviations as whiskers, and standard errors as rectangles. Asterisks (*) indicate antibiotics for which the differences between isolates from organic and conventional milk are statistically significant (*p* < 0.05). The abbreviations are the same as indicated in Figure 1.

Subsequent analysis focused on determining the minimum inhibitory concentrations (MIC) of selected antibiotics commonly used in human and veterinary medicine or suspected to be associated with tolerance development. Comparison of average MIC values for each tested antibiotic revealed no statistically significant interspecies differences (one-way ANOVA, *p*-values ranging from 0.986 to 0.072). Indeed, the tolerated concentrations of these therapeutics among *B. cereus*/*B. thuringiensis* and *B. mycoides*/*B. weihenstephanensis* were pretty similar. More interesting, however, is the comparison of the MIC values between strains from different sources, e.g., organic and conventional milk (Figure 2). It was found that *B. cereus s.l.* from organic milk exhibited statistically lower MIC values for all tested antibiotics except clindamycin. Substantial differences were observed for ceftriaxone (the average MIC value for strains from conventional milk was 2.05 times higher than for bacteria from organic milk). The difference was less pronounced for other strains, with MIC values for strains from conventional milk ranging from 1.17 times higher for chloramphenicol (a non-significant difference) to 1.6 times higher for amoxicillin with clavulanic acid (a statistically significant difference). An exception was rifampicin, for which isolates from organic milk exhibited higher average MIC values, though the difference was not statistically significant.

The differences in MIC values for clindamycin between organic and conventional milk were not significant for *B. cereus*/*B. thuringiensis*. However, for *B. mycoides*/*B. pseudomycoides*, the average MIC values for strains from conventional milk were significantly higher (1.46 times) compared to those from organic milk. However, there was a wide range of variability within the tested isolates. The obtained results do not appear alarming at first glance, as the average MIC values for meropenem, chloramphenicol, vancomycin, erythromycin, azithromycin, and rifampicin corresponded to *B. cereus s.l.* sensitivity to these drugs. However, the values of amoxicillin with clavulanic acid, ceftriaxone, and clindamycin fell within the intermediate range, while for penicillin, they indicated resistance. The MLST analysis of individual sequence types (STs) identified 16 STs containing multidrug-resistant strains (resistant to three classes of antibiotics) and 28 STs with isolates resistant to two classes of antibiotics (see Section 2.5). While resistance was generally observed in STs representing isolates from both conventional and organic milk, some multidrug-resistant STs were exclusive to either organic milk (e.g., ST-76 and ST-213) or conventional milk (e.g., ST-42, ST-377, ST-901, and ST-1180).

### 2.4. Toxicity

PCR screening revealed the presence of the *nheA* gene in all *B. cereus s.l.* tested strains. Additionally, 47% of isolates carried the *hblA* operon, while 39% displayed the presence of the *cytK* gene. Furthermore, no isolate contained the *ces* operon, which encodes enzymes involved in the non-ribosomal synthesis of the *B. cereus s.l.* emetic toxin. Comparison among species representatives showed that the *hbl* operon was noticeably more common in *B. mycoides*/*B. weihenstephanensis* than in *B. cereus*/*B. thuringiensis*. Conversely, *cytK* was most frequently observed in *B. cereus*/*B. thuringiensis*, though these differences were not statistically significant. Similarly, no significant differences in the frequencies of enterotoxin genes were found between strains from different sources. Chi-square test results indicated that the distribution of enterotoxin genes in *B. cereus s.l.* was random.

The Duopath Cereus Enterotoxins test which was developed to efficiently detect enterotoxigenic *B. cereus s.l.* strains reacts with the protein products of the *nhe* and *hbl* operons and allows for the assessment of which isolates can produce these toxins. In our study, we found that 194 isolates tested were positive for NHE biosynthesis, while 89 strains could produce HBL. These outcomes indicated that six strains carrying the *nhe* operon and five strains with the *hbl* operon did not demonstrate the ability to synthesize the NHE and HBL enterotoxins, or their production levels were below the detection threshold of the applied immunochromatographic tests.

**Figure 3:**
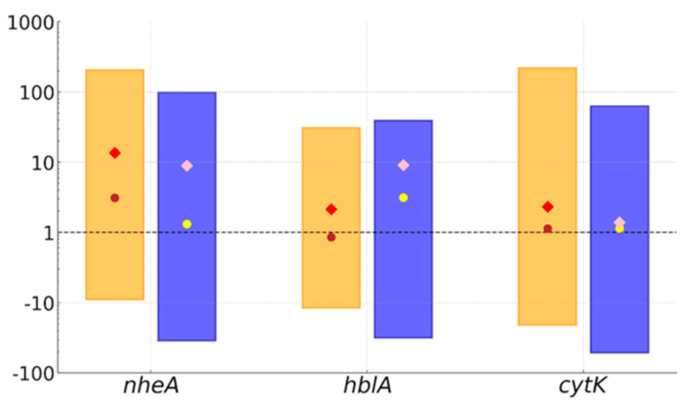
Variability in the relative expression levels of three enterotoxin genes of *Bacillus cereus* sensu lato among strains isolated from conventional milk (yellow boxes) and organic milk (blue boxes). Values are referenced against the expression levels of *nheA*, *hblA*, and *cytK* genes in the reference strain *B. cereus* ATCC 14579 (value of 1). Circles represent mean values, diamonds indicate medians, and boxes illustrate the range of values.

Discrepancies between PCR and immunochromatographic results prompted us to conduct quantitative PCR (qPCR) analyses to assess differences in the expression levels of the detected toxin genes. Relative expression analysis, using *B. cereus* strain ATCC 14579 as a control, revealed variability in the gene expression levels among the strains. A few strains exhibited expression levels 20–150 (*nheA*), 10–30 (*hblA*), and 50–200 times higher (*cytK*) compared to the control (Figure 3). For most isolates, expression levels ranged from 2–5 times lower to up to 5 times higher for all tested genes. Two strains with the *nheA* gene detected by PCR did not exhibit (within detection limits) expression of this gene, aligning with the immunochromatographic test results. Other isolates that yielded negative results in the Duopath Cereus Toxins tests showed gene expression near the detection threshold under the qPCR conditions despite the gene presence in conventional PCR.

### 2.5. Phylogenetic Relatedness vs. Antibiotic Resistance Profiles

**Figure 4:**
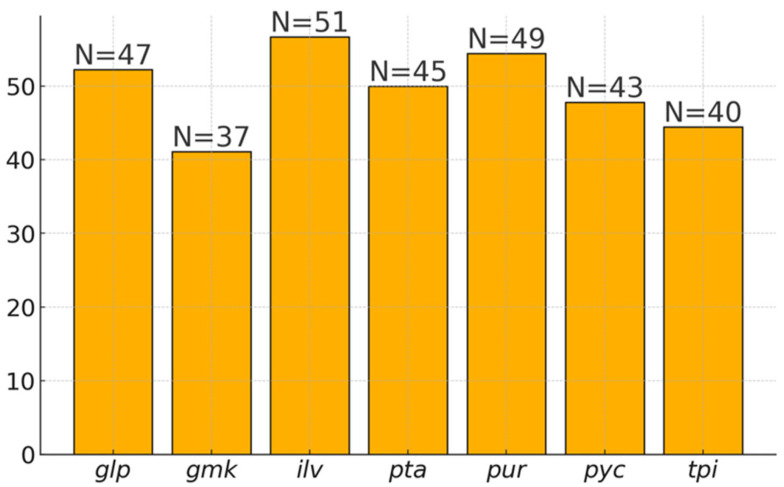
The percentage of unique alleles for the genes *glp*, *gmk*, *ilv*, *pta*, *pur*, *pyc*, and *tpi* among 90 identified sequence types of *Bacillus cereus* sensu lato. The values above the bars indicate the number of different alleles for each gene.

The phylogenetic relationship among the *B. cereus s.l.* isolates under study was reconstructed based on the nucleotide sequence comparison of seven housekeeping genes merged into a 2829 bp segment. Among the analyzed genes, a high level of polymorphism was observed. The *ilv* gene exhibited the highest variability (51 different alleles among 90 STs), while the *gmk* gene showed the lowest variability (37 alleles among 90 STs). The average level of polymorphism across the analyzed genes was approximately 50%, as shown in Figure 4. The allelic profiles of the isolates allowed their classification into 90 different sequence types (STs). Within the 2829 base pairs segment, 2237 sites were conserved (79%). The ratio of nonsynonymous to synonymous mutations, showing whether selective pressure was acting on the strains, was assessed at the value of 0.0296, indicating strong stabilizing selection that eliminates nonsynonymous mutations. Additionally, the Tajima Neutrality Test achieved a D value of 0.344 (positive). This D value suggested stabilizing selection and could indicate a subdivided population or an evolutionary bottleneck. Similar positive test values were observed when analyzing only strains from organic milk (D = 0.293) and conventional milk (D = 0.388).

**Figure 5:**
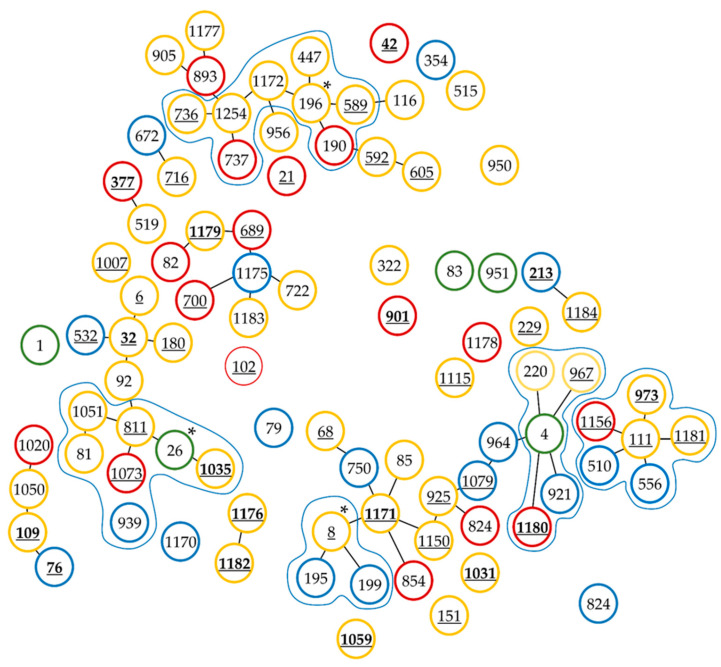
The population structure of *Bacillus cereus* sensu lato isolated from conventional and organic milk, determined using goeBURST analysis. Green circles represent sequence types (STs) corresponding to reference strains, blue circles represent STs of strains from organic milk, red circles represent STs of strains from conventional milk, and yellow circles represent STs represented by strains from both types of milk. Stars indicate central isolates within clonal complexes that have central isolates. Bold and underlined numbers represent STs corresponding to strains resistant to at least three classes of antibiotics, while underlined numbers correspond to STs of strains resistant to two classes of antibiotics.

In the goeBURST analysis, *B. cereus s.l.* isolates grouped into 19 clonal complexes (CCs), defined as their STs sharing at least four identical alleles (differing by a maximum of three alleles), along with 33 singletons. Among the CCs, one complex (CC-196) grouped eight STs, one (CC-26) included seven STs, the next (CC-111) grouped six profiles, CC-4 grouped five STs, two complexes grouped three ST profiles each (CC-1175 and CC-8). These data enabled the identification of clonal complexes with central profiles: CC-8 (STs: 8, 195, 199), CC-26 (STs: 26, 81, 811, 939, 1035, 1051, 1073), CC-196 (STs: 190, 196, 447, 589, 736, 737, 1172, 1254), and CC-1175 (STs: 722, 1175, 1183). Among the identified clonal complexes, we observed significant diversity in both the sources of isolates (each clonal complex encompassing at least 3 STs, including strains from both conventional and organic milk) and resistance profiles. In CC-811, one strain was multidrug-resistant, while two others were resistant to two antibiotics. In CC-4, one strain was resistant to three classes of antibiotics, and another to two classes. In CC-111, three isolates were resistant to two classes of antibiotics. Notably, most multidrug-resistant strains were found outside the main clonal complexes (Figure 5).

**Figure 6:**
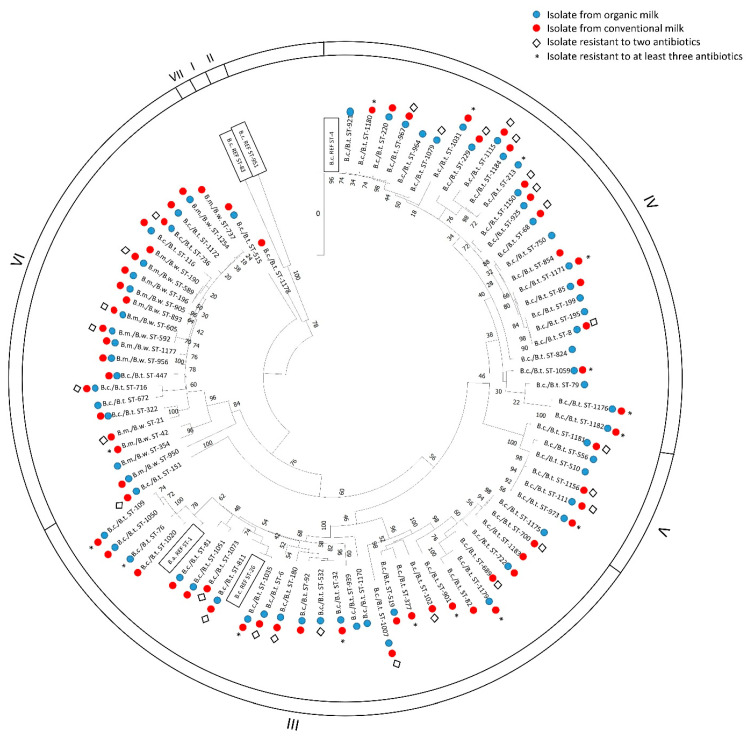
Genetic relatedness of *B. cereus s.l.* strains based on nucleotide sequence (2829 bp) comparison of seven housekeeping genes (*glp, gmk, ilv, pta, pur, pyc, tpi*), following the Multi-Locus Sequence Typing (MLST) scheme by [18]. The dendrogram was constructed using the Maximum Likelihood (ML) method, applying the GTR G+I model, selected based on the lowest BIC score, indicating optimal model fit for phylogenetic reconstruction with the presented data. The sequences of reference strains obtained from the PubMLST Database (pubmlst.org/bcereus) were included.

All *B. cereus s.l.* strains under study grouped into seven distinct clades (Figure 6). Clades I and II contained only reference strains, with the allele sequences corresponding to their sequence types obtained from the PubMLST database [18]. Clade III included strains with potentially the highest virulence, closely related to reference pathogenic and toxigenic strains (*B. anthracis* Ames and emetic *B. cereus* F4810/72). This clade contained over half of all isolates resistant to at least three different antibiotics, representing seven different sequence types. Additionally, *B. cereus s.l.* representing 10 other STs exhibited resistance to two different antibiotic groups. Clade IV, which included mesophilic *B. cereus*/*B. thuringiensis* isolates, also contained bacilli representing seven STs resistant to three antibiotic groups and another nine which were resistant to two drug groups, though the proportion of resistant strains here was nearly 30% lower than for bacteria grouped in Clade III. Meanwhile, Clade VI included *B. cereus s.l.* adapted to low temperatures, such as *B. mycoides*/*B. weihenstephanensis* and a few *B. cereus*/*B. thuringiensis* isolates. Only bacilli classified to one profile (ST-42) exhibited multidrug resistance, while isolates belonging to six ST profiles (STs: 21, 589, 592, 605, 716, 736) were resistant to two antibiotic groups. Although *B. cereus s.l.* showed marked polymorphism, psychrotolerant isolates generally grouped within Clade VI. However, the distribution of antibiotic-resistance genes did not correlate with the phylogenetic relatedness of isolates. Furthermore, there were instances where strains within a single ST exhibited different resistance profiles and tolerated doses of individual drugs.

## 3. Discussion

*Bacillus cereus s.l.* are widely distributed in the natural environment and food products, where they perform various functions [8,9,19,20,21]. These microorganisms exhibit complex taxonomy and a high degree of genetic diversity, among which only *B. anthracis*, which we did not find in our analyses, appears to be monophyletic and homogeneous. There is an ongoing debate on whether this entire group should be treated as a collection of separate taxa, a single polymorphic species, or a composite species comprising numerous ecotypes and pathotypes [22,23,24,25]. Since *B. cereus s.l.* phenotypic diversity mostly depends on the presence of large plasmids and their potential mobility, we decided to focus on the characteristics of individual bacteria and divided our milk isolates into mesophilic *B. cereus*/*B. thuringiensis* and psychrotolerant *B. mycoides*/*B. weihenstephanensis* [6]. These two groups are of primary interest due to their potential pathogenicity related to foodborne illness and frequent occurrence in active metabolic forms in food products [26,27] and even in water [7]. The risk associated with *B. cereus s.l.* is linked to their proliferation under refrigeration, toxins production, and increasingly growing antibiotic resistance [1,28,29].

We found that organic milk contained, on average, three times more *B. cereus s.l.* than milk from conventional farms. Interestingly, all conventional farms studied produced milk with very similar *B. cereus s.l.* contamination levels. The measures used for surface decontamination, teat cleaning, and general cleanliness on farms may help reduce bacterial levels and prevent their transfer into the final product. On the other hand, the use of natural bedding for cows, common in organic production, may significantly contribute to *B. cereus s.l.* contamination. The presence of *B. cereus s.l.* in milk was not surprising, especially given that previous studies have shown these bacteria in milk production environments and in raw and processed milk [30,31]. Equipment, machinery, and farm staff may also unintentionally contribute to bacterial spread. Additionally, on organic farms, animals were housed in a single large building without separate pens, which increased the likelihood of direct bacterial transfer between them. In contrast, cows on conventional farms were kept in individual pens. Therefore, it was essential to determine the extent to which these isolates pose potential health and epidemiological risks.

Qualitative assessment of antibiotic resistance among *B. cereus s.l.* indicated that fluoroquinolones, chloramphenicol, rifampicin, linezolid, gentamicin, and clarithromycin were the most effective, showing 100% efficacy. Interestingly, within the macrolide group, we observed the same result only for clarithromycin. Approximately 20% of isolates displayed intermediate sensitivity or resistance to erythromycin and azithromycin. The differences in efficacy among these drugs may be due to their chemical structures. Clarithromycin, more effective in our analyses, has an additional methyl group, enhancing its stability across a broader pH range. In contrast, azithromycin has a 15-member ring structure compared to the 14-member rings of clarithromycin and erythromycin. We also observed variability in the antibacterial efficacy of aminoglycosides. Gentamicin was effective against 100% of the strains, while streptomycin was effective against only 64% of the isolates. The remaining bacteria exhibited intermediate sensitivity to this aminoglycoside. The more complex structure of gentamicin likely facilitates its passage through the thick cell wall of Gram-positive bacteria, making it generally more effective than the simpler streptomycin. Overall, the prevalence of resistance in *B. cereus s.l.* aligns with previous studies [15,32,33], although these studies did not differentiate between organic and conventional food sources.

Beta-lactam antibiotics were the least effective, likely due to the widespread presence of beta-lactamases in these bacteria [34,35]. Treating *B. cereus s.l.* infections with tetracyclines may also present therapeutic challenges. Notably, we observed higher resistance and intermediate sensitivity to all tested antibiotics in isolates from conventional milk compared to organic milk, particularly for beta-lactams, macrolides, and tetracyclines (Figure 1).

Interestingly, no statistically significant differences were found between mesophilic *B. cereus*/*B. thuringiensis* and psychrotolerant *B. mycoides*/*B. weihenstephanensis* isolates. This suggests that the primary selective pressure arises from the milk environment rather than evolutionary factors or genetic relatedness among *B. cereus s.l.* isolates. While the presence of antibiotic-resistant *B. cereus s.l.* strains in milk poses potential therapeutic challenges, these data do not support evidence of tolerance development at progressively higher doses, often within subclinical concentration ranges. Nevertheless, this pattern highlights the role of selective pressure in driving antibiotic resistance, as previously demonstrated in studies comparing animal-derived foods, plant-based foods, and environmental samples [15].

To further investigate, we compared the range of minimum inhibitory concentrations (MICs) for selected antibiotics across *B. cereus s.l.* species and sources (Figure 2). Although no statistically significant differences were observed between taxonomic groups, substantial differences were evident between isolates from conventional and organic milk. Isolates from conventional milk displayed noticeably higher MIC values for all antibiotics except rifampicin. Significant differences were found for ceftriaxone, amoxicillin with clavulanic acid, and chloramphenicol.

Beta-lactam antibiotics, commonly used in therapy, may be inactivated by plasmid-encoded beta-lactamase enzymes that spread through horizontal gene transfer [36,37]. Interestingly, chloramphenicol, which is rarely used in veterinary practice due to severe side effects like anemia, showed higher tolerance levels among strains from conventional milk. The underlying mechanism for this remains unclear and warrants further investigation.

It is concerning that opportunistic *B. cereus s.l.* strains are gradually developing tolerance to increasingly higher doses of chemotherapeutics. This phenomenon is likely associated with conventional food production practices, where antibiotics are used more frequently in animal treatments and may be present in trace amounts in animal feed. Notably, although the animals from which our samples were collected were healthy and had not received antibiotics in the past 12 months, the emergence of resistance could result from the accidental introduction of already adapted bacteria or the acquisition of resistance genes by previously susceptible isolates. In *B. cereus s.l.*, even chromosomal gene transfer through horizontal gene transfer is possible. Findings from other studies, including analyses of the recombination-to-mutation ratio (r/m ratio), highlight the significant role of conjugation in the biology of *B. cereus s.l.* [38,39].

*B. cereus* and closely related bacteria are generally not regarded as serious health threats and are not typical targets for antibiotic therapy. However, they can cause infections in both humans and animals. In such cases, one-fifth of multidrug-resistant strains and over 40% of strains resistant to two classes of antibiotics could complicate therapeutic selection. Moreover, we must consider that *B. cereus s.l.*, as part of the microbiota in milk production environments, may inadvertently become a subject of natural selection. Even if they do not exceed the critical threshold for resistance, higher tolerated drug doses raise our concern and indicate that *B. cereus s.l.* should be viewed as a potential reservoir of resistance genes in the food industry. Additionally, these bacteria represent an exciting research model for studying antibiotic resistance and population structure formation processes.

Our analyses also included examining the population structure and potential enterotoxigenicity of *B. cereus s.l.* Genotyping results using the MLST scheme [18] revealed a high level of genetic diversity among the studied bacteria, consistent with previous reports [15,24,40]. Among the isolates studied, there was no one classified into groups I and II (apart from reference sequences from the PubMLST database), while groups IV (26 STs of wild-type isolates, primarily mesophilic *B. cereus*/*B. thuringiensis*), III (28 STs of wild-type isolates, mesophilic but more closely related and potentially more virulent *B. cereus*/*B. thuringiensis*), and VI (23 STs grouping cold-adapted *B. mycoides*/*B. weihenstephanensis*) were well represented, as shown in Figure 6. Psychrotolerant bacteria should be particularly well-suited to survive in milk, which is stored under refrigeration regardless of production type. Unsurprisingly, 65% of sequence types in group VI were found in both organic and conventional milk (compared to 54% of STs in group IV and 50% of STs in group III). The psychrotolerant nature of the milk isolates observed in our study aligns with the traditional grouping of *B. cereus s.l.* into group VI [22,24]. However, it was surprising that strains resistant to two or three antibiotic classes were evenly distributed across all phylogenetic groups. Thus, there is no clear correlation between the *B. cereus s.l.* taxonomic group or phylogenetic position and antibiotic resistance profile, and no correlation between clonal complexes (CCs) and resistance profile, reinforcing that the environment is the primary factor associated with resistance.

**Figure 7:**
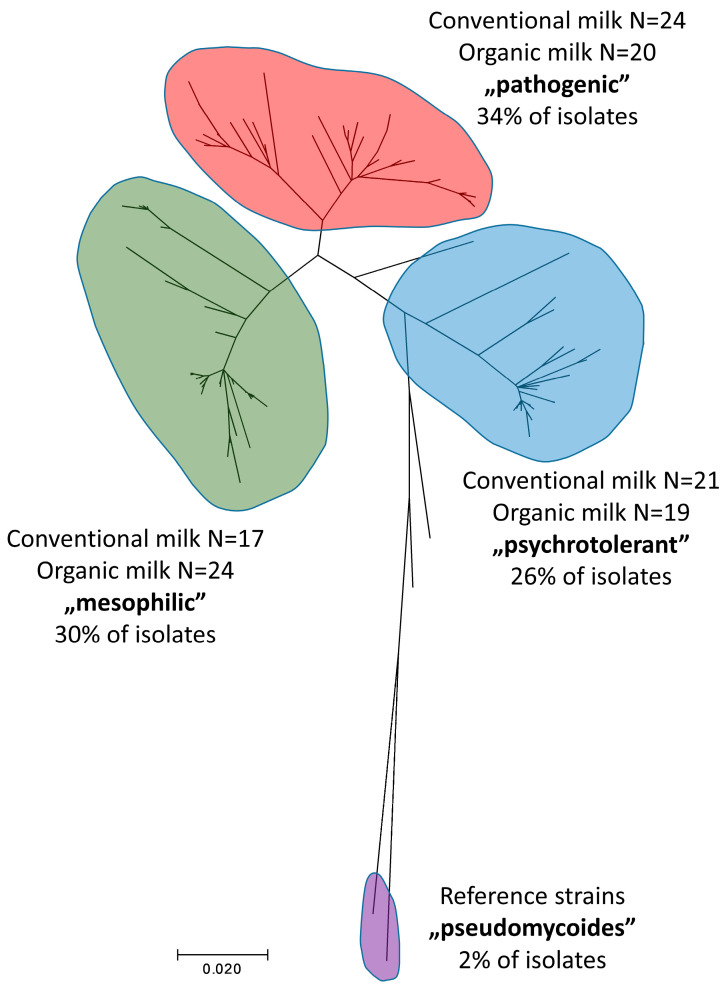
Phylogenetic relationship of *Bacillus cereus s.l.* reconstructed using MEGA 7 software (Maximum Likelihood method, GTR G+I model) based on concatenated sequences of seven housekeeping genes, following the MLST scheme in the PubMLST service [18].

MLST analyses revealed clear clustering of *B. cereus s.l.* strains based on their ecological properties. As shown in Figure 7, the analyzed strains form distinct psychrotolerant branches (26% of isolates with a relatively equal distribution of strains from both types of milk), mesophilic branches (30% of isolates with a moderate dominance of strains from organic milk), and pathogenic branches (34% of isolates with a slight predominance of bacteria from conventional milk). Only a few isolates displayed markedly different profiles, preventing their clear classification into any ecological group. These findings indirectly support the treatment of *B. cereus s.l.* as a highly polymorphic species with a range of ecotypes.

Additional genetic analyses, including the low dN/dS ratio, indicated that the studied *B. cereus s.l.* strains are under strong stabilizing selection. Similar results were obtained for organic and conventional milk strains, suggesting that selective pressures favor isolates adapted to tolerate antibiotics in both environments. It can be inferred that resistant *B. cereus s.l.* isolates may also be favored on organic farms, where occasional antibiotic treatment for sick animals is necessary. Even though milk from treated animals is not used during and after treatment, the selection of resistant strains still occurs, and these bacteria are transmitted between animals or persist in the environment as endospores. The positive result of the Tajima Neutrality Test further supports the hypothesis of natural selection. It indicates that the studied bacteria may have undergone a population bottleneck, particularly during antibiotic therapy, when only resistant strains survived. The calculated, relatively high recombination-to-mutation (r/m) ratios for both organic and conventional milk suggest that genetic recombination plays a significant role in maintaining the genetic structure of *B. cereus s.l.*, shaping genetic diversity. This may also contribute to the spread of antibiotic resistance in both bacterial populations studied. Relatively high r/m values counteract the bottleneck effect and stabilize selection, contributing to increased biodiversity [15]. Thus, the polymorphic nature of *B. cereus s.l.* in food is the result of an ongoing balance between stabilizing selection, supported by bacterial reduction during therapy, decontamination, and sanitation, and intense horizontal gene transfer (HGT) during periods between selective pressures from antibiotics.

The enterotoxigenicity of *B. cereus s.l.* is well-recognized. Yet high-quality processing procedures in the food industry significantly reduce the risk of severe contamination in food products [41]. It is important to emphasize that it is not the mere presence of bacteria in food products but rather their ability to effectively biosynthesize toxins that determines the health risk they pose [42]. In the qPCR analyses, we found that the toxigenic potential of the studied bacteria was comparable to the reference *B. cereus* ATCC 14579 strain, although certain isolates could biosynthesize toxins at much higher levels than the reference one. Individual strains capable of synthesizing specific enterotoxins at levels 10 to 200 times higher than the reference strain pose a significant threat if they enter the gastrointestinal tract of a consumer. Importantly, with one exception, the isolates exhibiting the highest toxin biosynthesis potential did not display multidrug resistance. This suggests that toxin synthesis capability may serve as an adaptation for more effective survival within the gastrointestinal tract. Additionally, diarrhea facilitates more efficient environmental dissemination of spores, contributing to evolutionary success. The lack of correlation between phylogenetic position or resistance profile and the potential for toxin biosynthesis suggests that enterotoxin formation may be a distinct adaptation for environmental survival, independent of and possibly even competitive with drug resistance or psychrotolerance.

## 4. Materials and Methods

### 4.1. Bacterial Isolation and Identification

Milk samples (1 L each) were collected from four dairy farms: two exclusively producing organic milk and two producing only conventional milk. From each farm, five independent milk samples were taken at three-day intervals. The samples were collected directly from farm tanks into sterile glass bottles and transported under refrigerated conditions to the laboratory for further analysis.

Bacteria were isolated from the milk samples using membrane ultrafiltration (Sartorius membrane filters, 0.8 μm pore size), following the method described by Bartoszewicz et al. [9]. Each sample was divided into 100 mL portions and pasteurized at 72 °C for 5 min to eliminate competing microflora and stimulate spore germination. To facilitate filtration, the samples were treated with trypsin and the detergent Triton X-100.

Prepared samples were passed through membrane filters, which were subsequently rinsed with 100 mL of sterile water to remove residual material. The filters were then aseptically transferred onto the surface of M.Y.P. agar (Mannitol-Egg Yolk-Polymyxin Agar, Oxoid, Basingstoke, UK) and incubated overnight at 30 °C.

Bacteria forming large, matte, or rhizoidal colonies (*B. mycoides*), as well as purple colonies on the MYP agar surface, were selected for further analysis. Hemolysis of these isolates was also assessed using Columbia SB agar supplemented with 5% sheep blood (Oxoid).

To confirm that the examined microorganisms belonged to the *B. cereus* group, 16S rRNA gene sequencing was conducted using primers described by Bartoszewicz et al. [43]. Moreover, the isolates were classified based on the distinctive characteristics of each bacterium, e.g., (i) mesophilic isolates (growing at 30 °C) with classic colony morphology were considered *B. cereus*/*B. thuringiensis*, (ii) while those forming regular or rhizoidal colonies and capable of growth at 7 °C but unable to grow at 43 °C and possessing the *cspA* gene with the characteristic 4-ACAGTT-9 sequence were identified as *B. mycoides*/*B. weihenstephanensis* [43].

### 4.2. Antibiotic Resistance

To assess *B. cereus s.l.* resistance profiles to selected antibiotics, the disk diffusion method was employed following the guidelines of the Clinical Laboratory Standard Institute [44] and the European Committee on Antimicrobial Susceptibility Testing [45]. Given the prevalent resistance to β-lactams and the varying sensitivity to other antibiotics, the analyses were extended to include the determination of the minimal inhibitory concentration (MIC) using E-test gradient strips (Oxoid). The resulting data were compared with resistance profiles for each strain. Statistical analyses are described in Section 4.6.

### 4.3. PCR and qPCR Analyses

PCR was used to amplify the following genes: (i) *16S rRNA*, *glp*, *gmk*, *ilv*, *pta*, *pur*, *pyc*, *tpi* for the isolates taxonomic characterization, (ii) of *cspA* (major cold-shock protein), typical for psychrotolerant isolates of *B. cereus s.l.*, (iii) *nheA*, *hblA*, *cytK* encoding enterotoxins in *B. cereus s.l.*), (iv) *ces*, encoding the emetic toxin synthetase, and (v) genes characteristic of pXO1-like (the *repX* gene) and pXO2-like (the *repA* gene) plasmids. DNA was extracted from 18-h bacterial cultures after cell lysis with lysozyme, followed by purification using the DNeasy Blood & Tissue Kit (Qiagen, Hilden, Germany) according to the manufacturer’s protocol for Gram-positive bacteria. The quality of the extracted DNA was assessed spectrophotometrically using NanoDrop 2000. Amplifications were performed with the Start Warm HS-PCR Mix (A&A Biotechnology, Gdańsk, Poland) in a GeneAmp 9700 thermal cycler (Applied Biosystems, Waltham, MA, USA), and the products were separated by electrophoresis on 1–1.2% agarose gels (Prona, Burgos, Spain). The primer sequences are provided in Table 1.

Differences in expression levels of selected genes from the enterotoxin-coding operons were analyzed via quantitative real-time PCR (*q*PCR). RNA was extracted from 18-h cultures incubated at 30 °C to support the growth of both psychrotolerant and mesophilic strains. RNA isolation was performed using the Total RNA Mini Plus Kit (A&A Biotechnology), according to the manufacturer’s protocol. The quality and purity of the RNA were verified with NanoDrop 2000 and 1.2% agarose gel electrophoresis (Prona). To ensure the absence of DNA residues, samples were treated with DNase (A&A Biotechnology). cDNA synthesis was carried out using the High Capacity cDNA Reverse Transcription Kit (Thermo Fisher Scientific, Waltham, MA, USA) in a GeneAmp 9600 thermocycler (10 min at 25 °C, 120 min at 37 °C, final heating 5 min at 85 °C). The resulting cDNA was used for qPCR, conducted in a StepOne Plus system (Thermo Fisher Scientific). The reaction mix included 10 μL RT 2× PCR Master Mix (A&A Biotechnology), 1 μmol of forward and reverse primers, and 2 μL of cDNA. Data analysis was based on the relative expression model, with *udp* serving as the endogenous control gene [46]. Primers used in the qPCR are listed in Table 1. Cut-off values were determined independently for each gene by comparison with a negative control. Relative gene expression was calculated using the Pfaffl model [47], with the typical *B. cereus* strain ATCC 14579 (American Type Culture Collection) serving as the positive control.

### 4.4. Toxigenicity Assessment

To evaluate whether the tested *B. cereus s.l.* strains were capable of producing non-hemolytic enterotoxin and hemolytic toxin, Duopath Cereus Enterotoxins immunochromatographic tests (Merck, Darmstadt, Germany) were applied. For the analysis, the strains were subcultured on nutrient agar to stimulate growth and confirm the purity of the cultures. Material from a single colony was used as inoculum for CGY medium (Oxoid). The prepared samples were incubated at 30 °C with vigorous shaking (200 rpm). After six hours of incubation, 100 µL of the culture was applied to cassette-based test strips. The tests were then incubated for 15–30 min, after which the results were recorded. Only samples that displayed a positive control band were included in subsequent analyses. This streamlined approach ensured accuracy and reliability in assessing the enterotoxigenic potential of the *B. cereus s.l.* strains while maintaining reproducibility and rigor in experimental conditions.

### 4.5. Phylogenetic Analysis

The evaluation of genetic relatedness among the studied strains allowed us to determine whether there was a correlation between their antibiotic sensitivity profiles and genetic diversity. For this purpose, the Multi-Locus Sequence Typing (MLST) was used according to the protocol by Jolley et al. [18]. This technique relies on sequencing seven housekeeping genes and establishing phylogenetic relationships based on sequence variations in individual genes. In the MLST *B. cereus s.l.* typing, the following genes were used: *glp* (glycerol uptake facilitator protein), *gmk* (guanylate kinase, putative), *ilv* (dihydroxy-acid dehydratase), *pur* (phosphoribosylaminoimidazolecarboxamide), *pta* (phosphate acetyltransferase), *pyc* (pyruvate carboxylase), *tpi* (triosephosphate isomerase). The primers used in MLST, and conditions of amplification are accessible at https://pubmlst.org/organisms/bacillus-cereus/primers (accessed on 12 October 2024). The PCR amplicons were purified using the PCR Clean-Up Kit (A&A Biotechnology) and then used as material for the sequencing PCR. The sequencing PCR products were purified from terminators with the Ex-Terminator kit (A&A Biotechnology), following the manufacturer’s protocol. An ABI3500 capillary sequencer (Applied Biosystems) was used for sequence reading.

Sequences were checked for accuracy and quality using Chromas Lite 2.0.1 software. Sequence alignment was performed with BioEdit version 7.0.1. The gene sequences (*glp*, *gmk*, *ilv*, *pta*, *pur*, *pyc*, *tpi*) were aligned using BioEdit 7.0.1 and imported into the MEGA 6 software, where the best-fit model for phylogenetic reconstruction was selected. A phylogenetic tree was generated using the Maximum Likelihood method with the GTR model and G+I corrections. Clonal complexes were identified using the BURST algorithm [48], available through the PubMLST portal (pubmlst.org/bcereus).

### 4.6. Statistical Analysis

To assess whether the concentration of *B. cereus* sensu lato in individual samples differed significantly, an independent samples Student’s t-test was applied. The MIC values for strains isolated from conventional and organic milk were averaged and analyzed using a one-way analysis of variance (ANOVA, *p* = 0.05) to determine the statistical significance of observed differences. Tukey’s *t*-test was applied to identify results that significantly deviated from the group mean. All statistical analyses were conducted using the Statistica 5.5 software (StatSoft, Tulsa, OK, USA). To evaluate whether the distribution of toxin genes among bacterial groups occurred randomly, a chi-square test was performed with a confidence level of *p* = 0.05.

Selective pressure on the housekeeping genes of *B. cereus* was assessed by calculating the dN/dS ratio based on MLST data. Sequences of seven housekeeping genes (*glp*, *gmk*, *ilv*, *pta*, *pur*, *pyc*, *tpi*) were analyzed to identify mutations and classify them as synonymous or nonsynonymous. The dN/dS analysis was performed using MEGA 6 software with the Nei-Gojobori model [49], which assumes an equal substitution distribution. For each gene locus, dN and dS values were calculated while accounting for the number of potential mutation sites. The average dN/dS value across all loci was then used to compare selective pressure between different groups of strains. Additionally, Tajima’s Neutrality Test was conducted to distinguish whether genetic changes in the studied material were driven by genetic drift (random events) or natural selection [50].

## 5. Conclusions

Our study demonstrated that *B. cereus s.l.* is commonly present in milk, with organic milk showing significantly higher contamination levels. Bacteria isolated from milk frequently exhibited resistance to beta-lactam antibiotics, including penicillins, amoxicillin, and amoxicillin combined with clavulanic acid. Moderate efficacy was observed with ampicillin and cephalosporins, while fluoroquinolones, phenicols, and rifamycins were the most effective in inhibiting bacterial growth. Isolates from conventional milk were more likely to exhibit multidrug resistance and tolerate significantly higher concentrations of antibiotics. This correlation between antibiotic resistance profiles and the source of the isolates highlights the impact of antibiotic use in animal husbandry on the selection of resistant strains, including opportunistic pathogens. Continuous surveillance of food contamination by antibiotic-resistant bacteria is essential to safeguard consumer health.

While organic food production reduced the development of antibiotic resistance among *B. cereus s.l.* in milk, it was associated with higher bacterial contamination levels, increasing the risk of foodborne illnesses. The presence of psychrotolerant isolates was particularly concerning, as it may compromise the safety and shelf life of refrigerated food products. Importantly, opportunistic bacterial species such as *B. cereus s.l.* may act as reservoirs of antibiotic-resistance genes, representing a potential threat to both public and veterinary health. These findings emphasize the need for targeted interventions, including stricter hygiene measures and sustainable farming practices, to minimize risks associated with bacterial contamination and antibiotic resistance in the food supply chain.

## Figures and Tables

**Table 1:** Primer sequences and the expected size of amplification products for PCR (genes *ces*, *cspA*, *glp*, *gmk*, *ilv*, *pta*, *pur*, *pyc*, *tpi*, *repX*, *repA*) and real-time PCR (genes *hblA*, *nheA*, *cytK*).

**Gene**	**Primer**	**Product Size [bp]**
*hblA*	F: AAGCAATGGAATACAATGGG R: AGAATCTAAATCATGCCACTGC	1154
*nheA*	F: TACGCTAAGGAGGGGCA R: GTTTTTATTGCTTCATCGGCT	499
*cytK*	F: GTAACTTTCATTGATGATCC R: GAATACTAAATAATTGTTTCC	505
*ces*	F: GGTGACACATTATCATATAAGGTG R: GTAAGCGAACCTGTCTGTAACAACA	1271
*cspA*	F: GAGGAAATAATTATGACAGTT R: CTT(C/T)TTGGCCTTCTTCTAA	160
*glp*	F: GCGTTTGTGCTGGTGTAAGT R: CTGCAATCGGAAGGAAGAAG	372
*gmk*	F: ATTTAAGTGAGGAAGGGTAGG R: GCAATGTTCACCAACCACAA	504
*ilv*	F: CGGGGCAAACATTAAGAGAA R: GGTTCTGGTCGTTTCCATTC	393
*pta*	F: GCAGAGCGTTTAGCAAAAGAA R: TGCAATGCGAGTTGCTTCTA	414
*pur*	F: CTGCTGCGAAAAATCACAAA R: CTCACGATTCGCTGCAATAA	348
*pyc*	F: GCGTTAGGTGGAAACGAAAG R: CGCGTCCAAGTTTATGGAAT	363
*tpi*	F: GCCCAGTAGCACTTAGCGAC R: CCGAAACCGTCAAGAATGAT	435
*repX*	F: CCATATCGTGCGATTCTTG R: GAGCAAATTCACTCGCATCA	583
*repA*	F: TAAATCTAAAAA(C/T)TC(A/G)AAAGCTG R: GGCATTCTGAAGAA(A/C/G)CCAAA	576

## Data Availability

The data will be available on request.

## Appendix A

**Table A1:** Proportion of antibiotic-sensitive, intermediate, and resistant strains among 200 *B. cereus s.l.* isolates from organic and conventional milk.

Antibiotic Group	Antibiotic	*B. cereus s.l.* (%)		
		Sensitive	Intermediate	Resistant
Penicillins	Penicillin	0	0	100
	Ampicillin	16.3	21.3	62.4
	Amoxicillin	0	5.7	94.3
	Amoxicillin with clavulanic acid	8.4	9.2	82.4
Cephalosporins	Ceftriaxone	8.4	48.2	43.4
Carbapenems	Meropenem	98.4	1.6	0
Macrolides	Erythromycin	78	18.6	3.4
	Azithromycin	81.2	13.2	5.6
	Clarithromycin	100	0	0
Tetracyclines	Tetracycline	69.8	19.8	10.4
Lincosamides	Clindamycin	93	5.7	1.3
Aminoglycosides	Gentamicin	100	0	0
	Streptomycin	63.9	36.1	0
Fluoroquinolones	Ciprofloxacin	100	0	0
	Levofloxacin	100	0	0
Oxazolidinones	Linezolid	100	0	0
Glycopeptides	Vancomycin	97.3	2.7	0
Phenicols	Chloramphenicol	100	0	0
Rifamycins	Rifampicin	100	0	0
